# The Use of *Drosophila* to Understand Psychostimulant Responses

**DOI:** 10.3390/biomedicines10010119

**Published:** 2022-01-06

**Authors:** Travis James Philyaw, Adrian Rothenfluh, Iris Titos

**Affiliations:** 1 Molecular Biology Graduate Program, University of Utah, Salt Lake City, UT 84112, USA; travis.philyaw@utah.edu; 2 Department of Psychiatry, Huntsman Mental Health Institute, University of Utah, Salt Lake City, UT 84108, USA; 3 Molecular Medicine Program, University of Utah, Salt Lake City, UT 84112, USA; 4 Department of Neurobiology, University of Utah, Salt Lake City, UT 84132, USA; 5 Department of Human Genetics, University of Utah, Salt Lake City, UT 84112, USA

**Keywords:** genetics, gene discovery, psychostimulant response, SUD, addiction, ADHD, ASD, *Drosophila*, cocaine, methamphetamine

## Abstract

The addictive properties of psychostimulants such as cocaine, amphetamine, methamphetamine, and methylphenidate are based on their ability to increase dopaminergic neurotransmission in the reward system. While cocaine and methamphetamine are predominately used recreationally, amphetamine and methylphenidate also work as effective therapeutics to treat symptoms of disorders including attention deficit and hyperactivity disorder (ADHD) and autism spectrum disorder (ASD). Although both the addictive properties of psychostimulant drugs and their therapeutic efficacy are influenced by genetic variation, very few genes that regulate these processes in humans have been identified. This is largely due to population heterogeneity which entails a requirement for large samples. *Drosophila melanogaster* exhibits similar psychostimulant responses to humans, a high degree of gene conservation, and allow performance of behavioral assays in a large population. Additionally, amphetamine and methylphenidate reduce impairments in fly models of ADHD-like behavior. Therefore, *Drosophila* represents an ideal translational model organism to tackle the genetic components underlying the effects of psychostimulants. Here, we break down the many assays that reliably quantify the effects of cocaine, amphetamine, methamphetamine, and methylphenidate in *Drosophila.* We also discuss how *Drosophila* is an efficient and cost-effective model organism for identifying novel candidate genes and molecular mechanisms involved in the behavioral responses to psychostimulant drugs.

## 1. Introduction

Psychostimulants with abuse potential, such as cocaine, amphetamine, methamphetamine, and methylphenidate, are drugs that increase central nervous system activity and arousal [1]. These drugs also elevate mood by activating the neural substrates associated with reward [1]. Stimulant drugs have a long history of use in humans and evidence suggests cocaine, in its unpurified form in the coca leaf, has been ingested for more than 3000 years [2]. More potent stimulants, such as processed cocaine and pharmaceutical amphetamine, are relatively new psychoactive substances. Processed cocaine was not available until the late 19th century, when it gained popularity as an additive in medicines, drinks, and cigarettes [2]. Pharmaceutical amphetamine and methamphetamine were not available until the early 20th century when they enjoyed a similar phase of popularity and broad application [3]. The recognition of abuse liability and harm potential of these substances led to their prohibition. While cocaine, amphetamine, and methamphetamine have all been regulated and restricted [2,3,4], their abuse continues to pose a global health concern, with an estimated of 47 million global users of cocaine and amphetamine-type stimulants as of 2019 [5]. The recreational doses of psychostimulant drugs that produce euphoria can lead to addiction and cognitive impairment, while overdoses can cause psychosis, circulatory collapse, and death. In contrast, lower doses produce cognitive enhancement and have a range of therapeutic uses [6,7]. These include treatment of the cognitive and behavioral impairments associated with attention deficit and hyperactivity disorder (ADHD) [8] and autism spectrum disorder (ASD) [9,10]. Despite successful clinical application, the molecular mechanisms mediating the different therapeutic actions of psychostimulant drugs remain unknown. Therefore, two main questions are the focus of continuing research (1) what molecular mechanisms are involved in the transition from recreational psychostimulant use to habitual maladaptive use, and (2) what are the molecular mechanisms that determine the therapeutic success of psychostimulant drugs for treating the symptoms of psychiatric disorders such as ADHD and ASD.

One approach to answering these questions is to take advantage of model organisms that are experimentally amenable and frequently used for their powerful genetics. Genes are a primary determinant of variation in behavioral responses to the addictive or therapeutic effects of psychostimulant drugs [11,12], and family studies show genes influence drug initiation [13] and addiction [14]. Addictions are among the most inherited psychiatric disorders, with an estimated genetic heritability component of ~72% for cocaine [15] and ~42% for non-cocaine psychostimulants [16]. Despite all the evidence from family studies, it has been challenging to identify the specific genes or genetic modifications that make an individual more susceptible to becoming a compulsive user [11]. Similarly, while it is known that genetic variation underlies differences in response to therapeutic drugs [17] little is known about the specific genes and molecules that impact therapeutic response to psychostimulants. Heterogeneity in the population and the subsequent need for a large sample size make it difficult to use human studies for identifying genes that impact psychostimulant response. In contrast to mammals, the model organism *Drosophila melanogaster* enables high-throughput analysis of behavior in assays that can scale to accommodate thousands of flies while supporting targeted genetic and molecular manipulations [18,19]. This review discusses how the model organism *Drosophila* can help elucidate the genetic basis of behavioral response to psychostimulant drugs to improve our understanding of human addiction and psychopathology.

### 1.1. Drosophila as a Model Organism to Study Addiction

*Drosophila* is a powerful model organism for studying the genetic and molecular basis of behavioral responses to drugs of abuse. A fast generation time, excellent genetic tractability, and a broad range of molecular tools enable precise spatiotemporal manipulation of gene expression, protein function, and cellular activity in *Drosophila* [18,20,21,22,23]. Around 75% of human disease-causing genes are conserved in flies [24], highlighting the translational application of *Drosophila* research [25]. Additionally, *Drosophila* is a valuable model for studying complex addictive disorders such as alcoholism [26]. Studies over the last 25 years have shown that flies express a range of alcohol-induced behaviors similar to those observed in intoxicated humans [27], and numerous genes isolated based on their involvement in *Drosophila* alcohol responses have helped identify corresponding genes that contribute to alcohol use disorder (AUD) in humans [28]. Additionally, the neurotransmitters important for mammalian alcohol-induced behaviors are also involved in *Drosophila* alcohol responses [29], including dopamine, which is required for reward in flies [26] and in humans [30]. The successful use of *Drosophila* to identify novel genes and mechanisms involved in human AUD provides the rationale for performing similar studies to investigate other addictive drugs. Psychostimulants increase dopaminergic signaling in flies as well as humans, but little is known about the genes, molecules, and pathways that influence the behavioral response to psychostimulants, making *Drosophila* a great model organism to answer these questions and provide valuable translational information.

### 1.2. Dopamine Is Central to the Molecular Mechanisms of Psychostimulant Response

**Figure 1:**
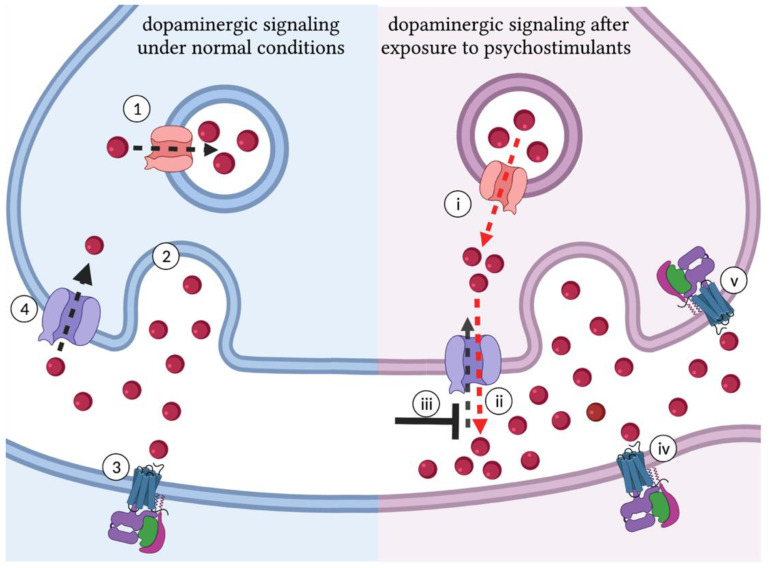
Dopaminergic signaling in the absence (blue background) and presence (pink background) of psychostimulant drugs. On the left, the steps of dopaminergic signaling represented include: (1) uptake of cytoplasmic dopamine by VMAT2 (black dashed line), (2) vesicular release of dopamine, (3) binding of neurotransmitter to post-synaptic dopamine receptor, and (4) reuptake of dopamine by the dopamine transporter (black dashed line). The increase in dopaminergic signaling caused by psychostimulant drugs is depicted on the right and include: (i) depletion of vesicular dopamine following interaction of VMAT with amphetamine-like psychostimulant drugs (red dashed line), (ii) amphetamine-induced reverse transport of dopamine (red dashed line), (iii) blockade of dopamine uptake (inhibition of black dashed line) by cocaine or methylphenidate, (iv) increased binding and activation of post synaptic dopamine receptor, and (v) increased binding and activation of pre-synaptic D2-like autoreceptor. Created with BioRender.com.

Experiences that benefit biological fitness, such as food or sex, are perceived as rewarding [31]. The association of salient stimuli with reward requires dopamine (DA), a monoamine neurotransmitter central to reward prediction and the reinforcement of motivated behavior [32,33,34]. Natural rewards such as food and sex, along with signals that predict natural rewards, are associated with phasic activation of distinct striatal dopaminergic neurons [35]. The bursts of dopaminergic signaling associated with natural rewards involve increased release of dopamine stored by vesicular monoamine transporter 2 (VMAT2) [36]. Dopamine released into the synaptic cleft binds D1-like (DRD1) and D2-like (DRD2) dopamine receptors, activating downstream G-protein coupled signaling cascades [37]. The plasma membrane dopamine transporter (DAT) removes dopamine from the synapse and indirectly reduces the activation of dopamine receptors [38]. Like natural rewards, drugs of abuse increase dopaminergic signaling, effectively hijacking the endogenous reward system (Figure 1) [39]. Cocaine and amphetamine-like psychostimulants activate the dopaminergic pathways associated with reward through interactions with the DAT [40] and VMAT2 [41] that increase synaptic dopamine [42,43,44,45]. Human brain imaging studies confirm that psychostimulants induce phasic firing of dopaminergic neurons in the brain regions associated with reward [46]. Additionally, this activity correlates with the reported euphoric effects of psychostimulants drugs [47].

### 1.3. Behavioral Responses to Psychostimulants

Psychostimulant administration in rodents leads to dose-dependent changes in motor behaviors including grooming, locomotion, dyskinesia, and seizures [48]. Flies exposed to cocaine exhibit dose-dependent changes in stereotyped behaviors that, as in mammals, range from increased grooming and hyperlocomotion to seizures and death [49]. Psychostimulants also influence social behavior in rodents, including courtship, where drug exposure promotes sexual behavior [50,51]. Flies exposed to vaporized cocaine or methamphetamine also display an increase sexual behavior and spend more time performing courtship displays [52]. These parallels demonstrate that *Drosophila* is a model with high face validity for studying the behavioral response to psychostimulants.

Dopaminergic signaling is central to psychostimulant-induced behavioral responses. In mammals, the locomotor activating effects of psychostimulants require increased dopaminergic signaling in the neural substrates associated with reward [53]. Disrupting psychostimulant interactions with DAT and VMAT2, or blocking the activation of downstream dopamine receptors in psychostimulant-activated circuits disrupts the effects of psychostimulant drugs. Wild-type mice, for example, respond to cocaine with increased locomotion, while mutant mice with a cocaine-insensitive dopamine transporter do not display an increase in locomotion after cocaine exposure [54]. Similarly, the VMAT2 inhibitor reserpine reduces amphetamine-induced increases in dopamine in mice [55], and DRD1 antagonists disrupt self-administration of methamphetamine in rats [56]. In flies, psychostimulants also increase dopaminergic signaling by binding to the *Drosophila* dopamine transporter (dDAT) and vesicular dopamine transporter (dVMAT) [57]. Disrupting dopamine synthesis in *Drosophila* with 3-iodo-tyrosine reduces the motor-activating effects of cocaine [58], blocking synaptic release of dopamine with the VMAT2 inhibitor reserpine disrupts the locomotor-activating effects of cocaine and amphetamine-like stimulants [59], and mutation of the D1-like dopamine receptor in *Drosophila* reduces self-administration of methamphetamine [60]. These experiments show that the molecular and pharmacological basis of psychostimulant-induced behaviors are conserved in flies and mammals, highlighting the mechanistic validity of *Drosophila* as a model organism for studying the genetic underpinnings of response to psychostimulant drugs.

## 2. Measuring Behavioral Responses to Psychostimulants in *Drosophila*

Exposure to psychostimulants impacts many behaviors, including locomotion, sleep, arousal, attention-like processes, learning, memory, and social interactions. These behaviors have been studied using assays that quantify motor behaviors, feeding behavior, and attention-like processes. Psychostimulant-induced changes in fly motor behavior produce motor-activating and motor-impairing effects. Assays corresponding to each of these responses allow measurement of sensitivity and sensitization to the activating or impairing effects of psychostimulants. Sensitization involves the development of increased sensitivity to the effects of a drug across subsequent exposures, and is associated with neuroadaptations that increase the salience of stimuli associated with drug reward [61]. In assays of feeding behavior, voluntary consumption of psychostimulant-containing food can be used to monitor changes in self-administration and preference over time. These behaviors are especially relevant to modeling the progression of drug-taking behavior observed in addiction, allowing interpretation of the reinforcing effects of each drug. Attention-like assays, on the other hand, can model the therapeutic application of psychostimulant drugs in rescuing impairments in flies exhibiting behavioral features of ADHD. Here, we discuss the advantages and shortcomings of these assays of motor activity, feeding, and attention-like processes, highlighting techniques that effectively decrease labor time, reduce subjectivity, and take full advantage of the fast generation time and prolific reproduction of *Drosophila.*

### 2.1. Assays of Motor-Activity

**Figure 2:**
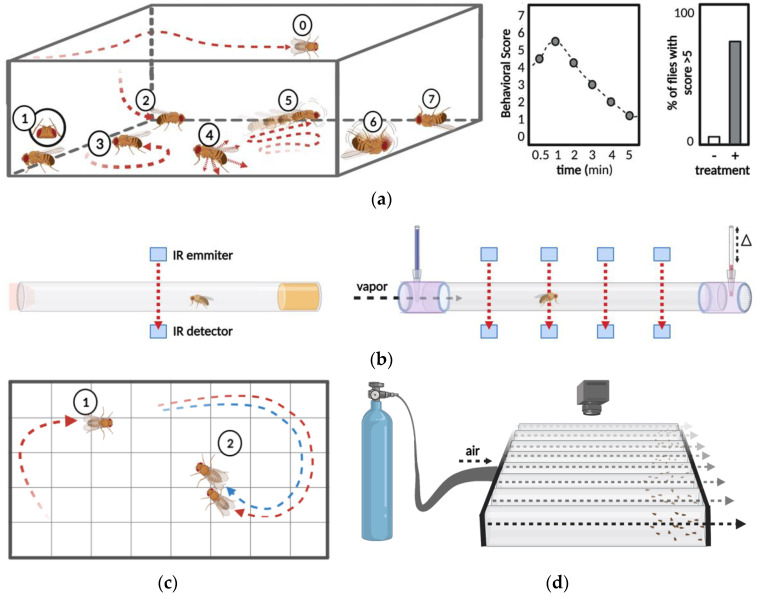
Assays for measuring the motor activating effects of psychostimulant drugs in *Drosophila*. (**a**) A representation of responses observed in the behavioral scoring assay at different concentrations of cocaine (left). Numbers correspond to the progression and severity of responses defined in the behavioral scale, which range from 0 to 7, as follows: (0) basal locomotion, grooming, and flight (1) decreased locomotion and increased grooming (2) simultaneous locomotion and grooming, proboscis extension, and loss of negative geotaxis (3) circling behavior and proboscis extension (4) leg twitching and erratic movements such as twirling and sideways or backwards locomotion (5) hyperkinesia and wing-buzzing (6) ataxia and seizures (7) akinesia and death. Graphs represent examples of how behavioral data are used to characterize the temporal dynamics of drug-induced responses (middle) as well as drug effect scores (right) which are represented here in an example comparing the percent of untreated flies (white bar) and drug-treated flies (grey bar) with behavioral score higher than 5, during a 1 min observation period. (**b**) *Drosophila* Activity Monitor (DAM) system cuvettes for measuring infrared (IR) beam breaks as a readout of locomotor activity. For chronic exposure experiments, drugs can be added to food (Left). In the newer *Drosophila* Activity Monitor 5M (DAM5M) each cuvette is intersected by 4 infrared beams, providing information on locomotion and position (right). Variations of the DAM allow acute administration of vaporized cocaine while measuring consumption and preference for individual *Drosophila*. In this setup, flies have access to two different feeding solutions in capillary tubes positioned at each end of the cuvette (right). Consumption is measured based on the displacement (Δ) of the solution, represented by the black dashed line. (**c**) A depiction of two different video-recorded locomotor assays with software driven automated analysis (1) the open field assay (2) courtship tracking assay (**d**) A schematic of the Repetitive Startle-induced Hyperactivity (ReSH) assay where locomotor response to mechanical stress is measured across eight tubes after flies are forced to one side with repetitive puffs of air. Created with BioRender.com.

One of the most obvious effect of psychostimulants is the activation of motor-related behaviors, an effect that is highly conserved across model organisms. Flies exposed to increasing concentrations of cocaine transition through phases of increased grooming behavior, hyperlocomotion, loss of negative geotaxis, stereotyped circling behaviors, erratic movements such as twirling, akinesia, seizures, and even death. One of the first ways this behavior was quantified was assigning a behavioral severity score using a behavioral scoring assay. Experimenters rated the activity of drug exposed flies from 0 to 7 using an ordinal scale of defined behavioral responses (Figure 2a). This behavioral scoring assay can also characterize the temporal dynamics of cocaine response by measuring latency to the peak of drug-induced behavioral effects and the time required to return to baseline. The peak response values recorded for individual flies can be used to generate a drug effect score, which represents the percent of flies that exceed an intoxication associated score during a defined observation period. In addition to defining the progression of cocaine-related behaviors in flies, this method allowed the analysis of how different doses or administration schedules impact sensitivity [49] and sensitization [62,63]. The development of behavioral sensitization is measured by performing repeated exposures to generate a time-course of drug effect scores. Exposures where the drug effect score is greater than the previous recorded score indicate the development of sensitization. The development of sensitization is a function of the interval after the first exposure, and for flies exposed to 75 μg vaporized cocaine, sensitization is only observed after exposure to a second dose 6–24 h after the initial exposure, but not before [62,63]. A similar interval is required for sensitization in mice, where cocaine-induced locomotion increases when the second dose is administered 24 h after initial drug exposure [64]. While the behavioral scoring assay can effectively measure sensitization, a critical psychostimulant response, scoring requires detailed observation of *Drosophila* behavior, is time-intensive, and subjective. One automated option that provides objectivity and can decrease the time spent scoring the motor activating effects of psychostimulants is the *Drosophila* activity monitor (DAM).

The DAM system is a high-throughput automated method of behavioral analysis that uses an infrared beam to detect motor activity [65]. Flies in the DAM system are individually housed in glass cuvettes transected by an infrared beam, and locomotor activity is analyzed by quantifying beam breaks (Figure 2b). The rate of beam breaks in the DAM measures activity as well as sleep and can be used to infer arousal state. Additionally, the software for processing DAM system data supports analysis of customized variables such as night time or day time activity, providing information on circadian patterns of response [66]. The DAM system has been used to measure the increases in locomotor activity that occur after injection [67], vaporization [68,69], or oral administration [52] of psychostimulant drugs. Every method of administration for cocaine or methamphetamine increases locomotor activity and decreases sleep in the DAM. The DAM also effectively measures sensitization, which is observed when a second dose of vaporized cocaine or methamphetamine is administered 6 or 10 h after the first dose, respectively [68,69]. The DAM also shows the wakefulness-promoting effects of methamphetamine in sleep-deprived flies, which sleep the same amount as flies that have not been sleep deprived, while sleep-deprived flies that are not given methamphetamine display significant rebound rest [52].

While each psychostimulant administration method produces measurable changes in behavior, it is important to consider tradeoffs in speed of delivery, precision of dosing, and how disruptive each method is to fly behavior. Drug-supplemented food provides the least control of speed and precision, but does not disrupt fly behavior. Drug injection is time consuming and disturbs normal fly behavior, but provides strict control of the dose. The FlyBong [68], designed to administer vaporized drugs in the DAM, provides a balance of speed and precision that enables simultaneous exposure for all flies without disrupting data collection, making it a valuable method for standardizing administration of psychostimulant drugs. Acute responses, in the hour following treatment, can be analyzed by recording beam break to measure activity rate (beam breaks/min) and number of activity peaks, where activity rate is at least double that of untreated controls [67]. Sensitization can be quantified based on relative changes in activity rate and number of peaks observed during successive psychostimulant exposures. For longitudinal assays, or experiments with orally administered psychostimulants, periods of inactivity can be measured to characterize the effects on sleep duration and architecture [52]. Regardless of drug administration method, the DAM system is a reliable and high-throughput method for measuring the locomotor response to psychostimulant drugs.

Multiple methods of drug administration can be combined with the DAM system, as demonstrated in a modified DAM where flies in each cuvette have access to capillary tubes filled with feeding solutions of 100 mM sucrose and 100 mM sucrose supplemented with methamphetamine [69]. Out of a range of concentrations, this experiment showed flies develop the strongest preference for 0.15 mg/mL methamphetamine, which is evident by the second day and remains stable for the remainder of the 7-day assay. Flies exposed to 75 μg vaporized methamphetamine in the FlyBong display increased locomotor activity after exposure, this activity more than doubles for flies subjected to a second exposure 10 h later. Transferring these sensitized flies to the capillary feeding DAM changes the outcome for preference, which no longer develops during the 3-day period of measurement [69]. These results are similar to those observed in rats, where methamphetamine self-administration is reduced after the development of sensitization following injections of methamphetamine [70]. Reciprocally, flies that develop preference in 3-day capillary feeding DAM performed prior to vaporized methamphetamine exposure in the FlyBong do not develop sensitization after the second 75 μg dose [69]. The relationship between preferential drug-consumption and locomotor sensitization, two behaviors with face validity for modeling addiction, demonstrates how seemingly unrelated endophenotypes can be intertwined at a mechanistic level. Accordingly, uncovering the molecular mechanisms involved in a simple response like sensitization can be informative about the molecular mechanisms that influence preferential self-administration.

While the constrained analysis of locomotion in the DAM is a reliable automated method for measuring the motor activating effects of psychostimulant drugs, some behaviors cannot be measured within the interior of a glass cuvette. Several assays that address this issue allow measurement of locomotion in freely moving *Drosophila*. Locomotor responses to psychostimulant drugs in adult or larval *Drosophila* can be measured by video recording responses in an observation chamber. Adult *Drosophila* [71] and 3rd instar larvae [72] fed cocaine display significantly increased rates of locomotion. These studies quantify locomotion rate by placing the observation chambers over a grid and counting the lines crossed during an observation period (Figure 2c). The availability of 2D-tracking and video processing software support automated processing of video recorded assays, which reduces the time required for analysis and limits interpretive bias [73,74,75].

Psychostimulant responses in *Drosophila* have also been studied by analysis of repetitive startle-induced hyperactivity (ReSH), which is based on locomotor response to recurrent puffs of air [76]. Air puffs are delivered to 8 groups of flies using the “puff-o-mat”, a device based on a similar apparatus used for the delivery of ethanol vapor (now appropriately known as the “booze-o-mat”) [77,78]. Recordings of the assay are analyzed using a custom software that measures changes in fly position, acceleration, velocity, and trajectory following mechanical stress from puffed-air (Figure 2d) [76]. Activity peaks immediately after the startle stimulus, and then slowly returns to baseline. Measurement of ReSH in untreated control flies shows large increase in velocity followed by a slow decay toward baseline [76]. Cocaine leads to a dose-dependent decrease in ReSH, reducing peak velocity after startle and reducing the decay period. Interestingly, the state of arousal measured during ReSH differs from the locomotor arousal associated with spontaneous activity quantified in the DAM, and is inversely impacted by cocaine, which reduces rather than increases locomotor activity [76].

### 2.2. Assays of Motor-Impairment

While psychostimulants activate some motor behaviors, they can impair others. This is the case for negative geotaxis [58] and some aspects of courtship behavior [52]. One possible explanation for this impairment might be the complexity of these behaviors. Negative geotaxis is a complex behavior that requires flies to integrate sensory information about light as well as gravity [79], and is even affected by electromagnetic fields [80]. Similarly, *Drosophila* courtship is a complex social behavior, with successful copulation requiring the integration of audible [81] visual [82] olfactory [83] and gustatory [84] stimuli. For some *Drosophila* behaviors, such as response to visual stimuli, optimal responses occur within a specific range of dopaminergic activity and are disrupted when dopamine levels are outside of that range [52]. It is likely that psychostimulant-induced increases in dopamine similarly impair negative geotaxis and courtship behaviors, which require complex processing and integration of sensory information across several modalities to mount an appropriate response.

**Figure 3:**
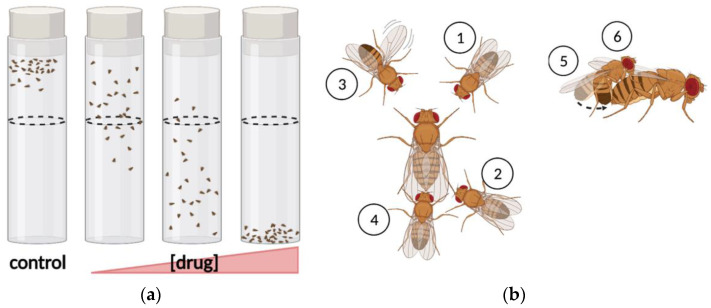
Assays for measuring motor-impairing effects of psychostimulants in *Drosophila* (**a**) Representation of climbing behavior in the negative geotaxis assay. Untreated flies are located at the top of the tube, while psychostimulant exposure disrupts climbing behavior in a dose dependent manner. (**b**) Depiction of the courtship stages quantified in the courtship behavior assay. Numbers represent successive steps in the courtship process, where 1–4 (left) depict a single male fly performing four sequential courtship displays: (1) orientation (2) tapping (3) wing-song (4) licking. Step (5) and (6) portray copulation attempts (mounting behavior) along with successful copulation, respectively. Recorded video data are used to quantify the number and duration of courtship behaviors. Created with BioRender.com.

Startle-induced negative geotaxis (SING) was one of the first behavioral assays developed in *Drosophila*, dating back to Seymour Benzer’s experiments in the 1960s [85]. Benzer joined two test tubes, forced the flies to the bottom of the enclosure, and scored the portion of flies that move from the proximal tube to the distal tube within a brief recovery period. SING condenses the analysis of motor response for multiple flies to one value, defined by the proportion of flies that make it past a defined height along the tube. The efficient quantification of this assay makes it amenable to automation: recordings can be processed with video analysis software. Additionally, this assay is easily scaled up allowing simultaneous measurement of locomotor responses across several groups of flies, exploiting the economy of scale that *Drosophila* offer. Untreated control flies quickly climb to the top of the tube, whereas flies exposed to cocaine exhibit dose-dependent impairments in negative geotaxis [58] (Figure 3a). Several variations of this assay have been developed, and provide similar measures of intoxication. A drug effect score can be determined with consecutive climbing assays based on the average number of flies remaining at the bottom of each cylinder after exposure [86], and acquisition as well as analysis can be automated using pictures captured from beneath each cylinder [87] to increase measurement speed and consistency. Alternatively, both the “startle” step and analysis of climbing behavior have been automated in the rapid iterative negative geotaxis (RING) assay, which allows measurement of average distance traveled, increasing the resolution and dynamic range of behavioral analysis [88]. In summary, assays of climbing behavior offer a high-throughput measurement of sensitivity and sensitization with automated analysis.

In contrast to the simplicity of measuring negative geotaxis behavior, the quantification of *Drosophila* courtship requires analysis of a series of distinct behaviors. An established method for studying these behaviors in males is the courtship behavior assay (Figure 3b). This assay involves quantification of courtship activities that follow a stereotyped order which includes orientation, tapping, wing vibration, licking, attempted copulation, and copulation [89]. A male is observed in a behavioral chamber together with one or more females to quantify the time it takes to initiate courtship, the number and duration of each courtship behavior, the number of copulation attempts, and the duration of successful copulation events. The copulation success rate is quantified in an index generated by recording pairs of *Drosophila* and measuring the proportion that successfully copulate [52]. While this assay is not high-throughput, it has the advantage of simultaneously allowing measurement of the motor activating and impairing effects of psychostimulants. Flies fed methamphetamine have a decreased latency to initiate courtship, and spend more time performing steps of the behavioral-courtship sequence that precede copulation. This increase in both speed of initiation and percent of time spent courting are associated with increased sexual arousal. In contrast, methamphetamine reduces the percentage of flies that successfully copulate while increasing the latency to copulation in flies that do, demonstrating how methamphetamine disrupts neural processes involved in initiating and sustaining the motor behaviors required for copulation [52].

### 2.3. Assays of Consumption and Preference

**Figure 4:**
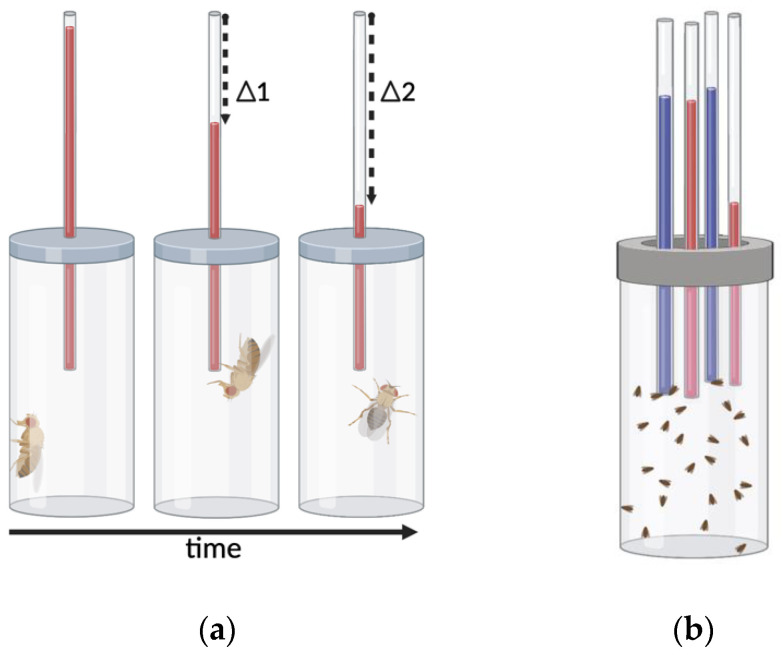
Assays used to measure psychostimulant consumption and preference in *Drosophila* (**a**) A single-fly assay of voluntary consumption where the rate of consumption is determined by monitoring displacement of the solution over time. The black dashed lines represent the cumulative change in volume recorded at the first and second measurement timepoints. (**b**) Flies housed in a Multiple Capillary Feeder (MultiCAFE) Assay with access to two different feeding solutions, depicted here as red and blue. The consumption of each solution over time is determined by monitoring the displacement the meniscus in each capillary. Data are used to characterize rate of consumption, cumulative consumption, and preference. Created with BioRender.com.

Assays of voluntary consumption are indispensable techniques for studying addiction and can be used to measure increases in self-administration over time. The CApillary FEeder (CAFE) assay [90] is a reliable feeding assay that allows continuous monitoring of consumption of liquid food from capillary tubes. Results characterize cumulative consumption, average consumption and consumption rate (Figure 4a). Furthermore, the CAFE can be modified to administer two different feeding solutions in a two-choice assay of consumption enabling analysis of preferential consumption (Figure 4b). Findings using the CAFE show that the development of amphetamine preference in *Drosophila* is concentration-dependent. Flies given the choice between a sucrose solution and a sucrose solution containing 1 mM amphetamine develop preference for the amphetamine containing solution by the first measurement timepoint at 24 h [91]. When the amphetamine concentration is increased to 10 mM this effect is not observed, and flies prefer the sucrose solution instead [91]. A similar experiment comparing daily consumption of two sucrose solutions, where one is supplemented with methamphetamine, showed that flies develop preference for 1 mM methamphetamine after one day while always avoiding 10 mM [60]. In contrast, flies did not develop preference for cocaine at any concentration (10 uM, 100 uM, 1 mM, 10 mM) during a four day CAFE, but avoided the two higher concentrations. The CAFE can also be used to identify the genes that influence cocaine and methamphetamine consumption, which has been investigated in large scale assays with hundreds of genetically distinct lines derived from the *Drosophila* Genetic Reference Panel (DGRP) [92,93]. Additionally, variations such as the FlyCAFE [69] allow measurement of locomotion in tandem with consumption at single fly resolution, and enable additional administration of vaporized drug [68]. Together, this combination of techniques allow simultaneous analysis of drug consumption, preference, and drug-induced locomotion, being a valuable tool for modeling the changes in consumption observed during the development of addiction.

### 2.4. Attention-like Processes

**Figure 5:**
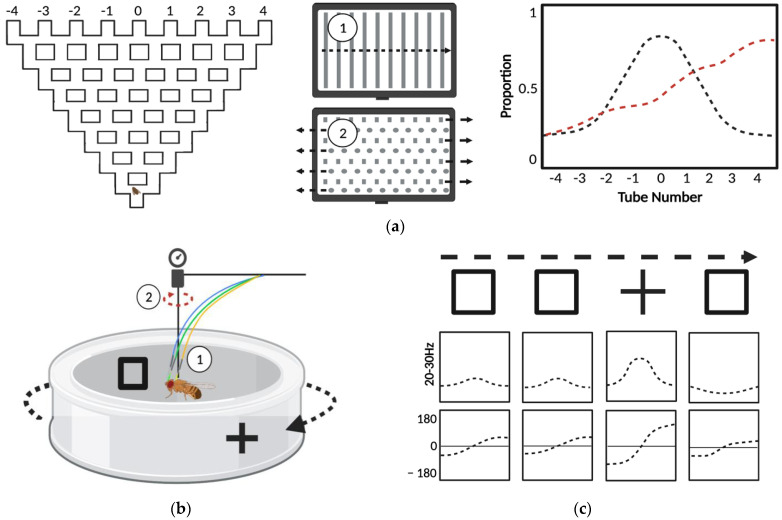
Assays for measuring attention-like processes in *Drosophila* (**a**) Depiction of a fly at the first of 8 successive choice points in a optomotor maze with nine possible outcomes. The bottom of the maze is clear, allowing presentation of visual stimuli by placement on top of monitor, here the maze is depicted in the absence of visual stimuli. The numbers across the top represent collection tube designations, and the values used for assigning tube score. A representation of two different patterns of moving visual stimuli (middle) that can be placed under the Y-maze to measure optomotor response (1) or optomotor distraction (2). Line graph depicting the proportion of flies in each collection tube (right) for an assay performed in the absence of visual stimuli (black dashed line) and an optomotor response assay involving presentation of a moving-grating pattern (red dashed line). (**b**) Ilustration of a tethered fly positioned in a visually programmable flightless arena where responses to visual stimuli are determined based on (1) electrophysiological recordings of brain activity and (2) measurement of physical response using a torque meter. (**c**) Depiction of visual-stimuli sequentially presented in a panorama during a closed-loop assay above representative responses to novel and non-novel visual stimuli that move across a tethered fly’s field of view, which is centered at the middle of each pane. The top row of boxes depicts 20–30 Hz Low Field Potential recordings of neural activity while the lower row represents the magnitude of torque responses. Created with BioRender.com.

Attention to specific stimuli is a process that requires selection and suppression of incoming sensory information. In tests of visual attention in humans, patients with ADHD display deficits in attentional selection [94], increased impulsivity, and distractibility [95]. Psychostimulants improve selective attention in patients with ADHD, and low doses also improve cognitive functioning in non-ADHD subjects [8]. While attention is more difficult to define in animals, many behaviors allow the measurement of attention-like processes. In *Drosophila*, attention-like processes have been studied by measuring responses to visual stimuli in the optomotor maze (Figure 5a) [96] and with flies suspended in a flight arena [97]. The floor of the optomotor maze is transparent and visual stimuli are introduced by placement over a monitor. Optomotor response is determined in single flies based on locomotion following the introduction of a uniform field of moving visual stimuli, and distractibility is measured based on the change in locomotion following the introduction of competing visual stimuli that move in opposite directions [52]. In flight arena experiments, attention-like processes are studied by measuring responses to visual stimuli in controllable panoramic [98] or programmable visual environments [99] that allow manipulation of visual stimuli presented to a single fly positioned within the arena (Figure 5b). In closed-loop flight arenas, fly behavior directly impacts the observable environment, creating a “closed” feedback loop, or a virtual flight simulator [100]. In contrast, flies in the flightless open-loop experiments have a limited range of motion and their responses do not influence visual stimuli, which rotate at a constant frequency independent of fly behavior (Figure 5c). In these experiments, shifts in visual fixation associated with presentation of novel stimuli are reflected in fluctuations of activity in 20–30 Hz local field potentials, as measured with a recording electrode [101]. Dynamics of 20–30 Hz have been used characterize “attention span” by measuring responses to novel stimuli, and responses to conflicting stimuli in assays of distractibility [101]. These attention-like processes can also be studied by measuring the intensity of torque that occur in response to visual stimuli. Both locomotor and electrophysiological measurements have been used to characterize behavior in mutant flies that have impairments in attention-like processes.

## 3. Studying the Therapeutic Use of Psychostimulants with *Drosophila*

ADHD and ASD are complex diseases that impact arousal, attention, and sleep. Dopaminergic dysfunction is a common characteristic of ADHD and ASD that contributes to cognitive and behavioral impairments [102,103]. Psychostimulant drugs improve some of the deficits seen in ADHD in humans [104], as well as in fly models of ADHD-like behavior [105], which we review in this section. We discuss how *Drosophila* responses to psychostimulant drugs have been used to characterize the behavioral effects of ASD-related mutations from human patients with dopaminergic dysfunction. The results of psychostimulants studies in *Drosophila* the models of ADHD and ASD are also discussed in Table 1.

### 3.1. Attention Deficit Hyperactivity Disorder (ADHD)

Hyperactivity and reduced sleep are common behavioral symptoms of ADHD [118,119,120]. Several mutant fly lines also exhibit hyperactivity and reduced sleep, making them face-valid models of ADHD-like behaviors. Among these are mutant lines for *Drosophila* orthologs of the *dopamine transporter* (*DAT*), *latrophilin-3* (*LPHN3*), and *neurofibromin-1* (*NF1*). These three genes are associated with ADHD in humans, and were chosen to test whether disruption of the fly ortholog would mimic ADHD-like behaviors in *Drosophila* [105]. The dopamine transporter was selected through database mining by searching for *Drosophila* genes with phenotypic descriptors related to hyperactivity, excitability, or attention. This search produced a list of 78 genes, with 69 conserved in humans. Five of those conserved genes, including *DAT*, were also identified in a list of 91 human ADHD-associated genes. The *Drosophila DAT* null mutant *DAT^fmn^* was identified based on its sleep phenotype—*fmn* stands for *fumin*, Japanese for sleepless [121]. Observation of *DAT^fmn^* flies using the DAM system showed hyperactivity and reduced sleep, with hyperactivity exacerbated in the absence of light, e.g., at night, but also during the subjective daytime when housed in dark:dark conditions. Feeding *DAT^fmn^* flies methylphenidate [105] or amphetamine [111] rescued hyperactivity and sleep loss. This study highlights how disruption of *Drosophila DAT*, the orthologue of a well-known ADHD risk gene highly represented in human GWAS studies, recapitulates two common symptoms of ADHD observed in humans that can be rescued by psychostimulant drugs used to treat the symptoms of human ADHD.

While *DAT* knockout is useful for modeling the effects associated with loss of function, there is significantly more variability in the *DAT* mutations that contribute to the prevalence of ADHD in humans [122,123,124]. Experiments in cell culture show that many psychiatric disorder-associated *DAT* variants exhibit heterogenous molecular phenotypes, including differences in DA uptake kinetics, reverse transport, and altered binding to psychostimulant drugs [125]. *Drosophila* is an efficient model for studying the effects of human *DAT* mutants in vivo and have shown that *DAT* mutations associated with early-onset Parkinson’s [126] as well as ASD [107] lead to impairments in motor behavior. *Drosophila* is a feasible system for performing similar experiments to unravel the behavioral and molecular nature of *DAT* variants associated with ADHD, offering a practical model to identify the molecular mechanisms involved in response to psychostimulant drugs.

In contrast to DAT, the influence of the G-Protein coupled receptor and cell adhesion protein Latrophilin-3 on dopaminergic signaling is poorly understood. *LPHN3* is an ADHD-risk gene that was identified in a linkage study based on a prevalence of ADHD with large generational families of an isolated population in the Paisa region of Colombia [127]. *LPHN3* variants that lead to haploinsufficiency are associated with the development of ADHD in humans [128]. While patients with *LPHN3* risk alleles respond to stimulant medication, a molecular mechanism linking reduced *LPHN3* expression to dopaminergic dysfunction has not been unraveled in human studies [128]. The behavioral effects of knockdown of the *LPHN3* fly ortholog have also been studied in *Drosophila* using the *GAL4/UAS* binary expression system. This method uses a cell type or tissue-specific enhancer to drive expression of the yeast transcriptional activator GAL4. Transgenes coupled to the GAL4 upstream activation sequence (*UAS*), are expressed wherever GAL4 is transcribed. The pan-neuronal driver *elav-GAL4* was used to drive expression of *UAS-Cirl-RNAi,* the single *Drosophila* orthologue for the *Latrophilin* family of genes. This reduced complexity can be beneficial when attempting to characterize a gene’s molecular function. In mammals, the effects of mutating a gene that is a member of a multi-gene family can be difficult to observe because of genetic redundancy. In flies, the reduction in gene copy number makes characterizing loss of function phenotypes more straightforward. Neuronal knockdown of the *Drosophila* orthologue of *LPHN3* was sufficient to produce the hyperactivity and decreased sleep associated with dopaminergic dysfunction [105]. As with the *DAT* null mutants, hyperactivity was more pronounced at night and could be exacerbated during the day by turning off the lights. Both the hyperactivity and sleep loss were rescued by feeding *Drosophila* methylphenidate. Tyrosine hydroxylase staining in fly brains showed that knockdown of the *Drosophila* orthologue of *LPHN3* did not alter the distribution or survival of dopaminergic neurons, indicating that *LPHN3* regulates dopaminergic signaling directly [105]. The role of *LPHN3* in dopaminergic signaling and variation in response to psychostimulant drugs is poorly understood. *Drosophila* is a useful model for studying neurotransmission [129,130] and offers a flexible system for the future identification of the molecular mechanisms that lead to altered amphetamine and methylphenidate responses in models of *LPHN3* disruption.

*NF1* is associated with the autosomal dominant disease neurofibromatosis type I. Neurofibromatosis type I is a multi-system disorder involving tumors of the nervous system that lead to complications such as eye disease, musculoskeletal disorders, and epilepsy, among others. In addition to the physical impairments associated with neurofibromatosis type I, most patients display cognitive deficits [131], including symptoms of ADHD [132]. In some patients, these ADHD symptoms are improved by treatment with methylphenidate [133]. To investigate whether loss of *NF1* would lead to ADHD-like behavior in flies, *NF1* was knocked down in all neurons of the *Drosophila* by using *elav-GAL4* to drive expression of *UAS-NF1-RNAi*. Knockdown of *NF1* in *Drosophila* neurons induced a hyperactivity and sleep-deficiency phenotype, with a noticeable increase in nighttime hyperactivity. Feeding *NF1* knockdown flies methylphenidate rescued both hyperactivity and sleep dysregulation [105].

The psychostimulant-mediated rescue of hyperactivity as well as sleep-deficiency phenotypes observed for *DAT*, *LPHN3*, and *NF1* orthologue mutants in the DAM system indicate that (1) hyperactivity along with reduced sleep is an endophenotype for ADHD in flies, (2) flies are a model with mechanistic validity for studying ADHD such that genes whose disruption leads to ADHD in humans also produce ADHD-like behavior in flies, and (3) flies are a model with predictive validity for studying ADHD since the psychostimulant drugs that reduce the behavioral symptoms of ADHD in humans also reduce ADHD-like impairments in flies. While *DAT*, *LPHN3*, and *NF1* all respond to treatment with methylphenidate, the molecular basis for the psychostimulant-mediated rescue of ADHD-like behaviors is unknown. Targeted knockdown of *DAT*, *LPHN3*, or *NF1* specifically in *Drosophila* dopamine neurons or subsets of dopamine neurons might help unravel how mutations with opposite effects on dopaminergic signaling can display similar response to psychostimulant drugs such as methylphenidate.

In addition to the locomotor signature observed with ADHD-like changes in sleep and hyperactivity, *Drosophila* can model more complex ADHD-like symptoms associated with dysregulation of attention-like processes [101]. Humans patients with ADHD have deficits in visual attention [94,134,135] that are improved by treatment with psychostimulants [134]. The *Drosophila* memory mutant *radish*^1^ also displays impaired responses to visual-stimuli in the optomotor maze and experiments of tethered flight [101]. Wild-type flies traveling through the optomotor maze turn in the direction of moving visual stimuli, an optomotor response that is absent in *radish*^1^ mutants flies [101]. This deficit in response is not a result of visual impairment, as *radish*^1^ mutants are successful in operant visual learning [135]. Methylphenidate rescues optomotor response in *radish*^1^ mutants, generating responses to visual stimuli similar to those observed for wild-type flies in the optomotor maze [101]. In addition to the altered locomotor response to visual information in the optomotor maze, *radish*^1^ mutants flies also exhibit altered brain activity in response to visual stimuli. Normally, flies display an increase in 20–30 Hz local field potentials (LFP)—observed by brain recordings—in response to visual stimuli. Electrophysiological recordings from flies suspended in a flightless arena show that flies repeatedly shown the same shape will display an increase in 20–30 Hz response when presented a novel shape. In contrast, *radish*^1^ mutants have a diminished 20–30 Hz response to novel visual stimuli. Treatment with methylphenidate also rescues the 20–30 Hz response to novelty in *radish*^1^ [101].

The impairment of visual attention-like processes observed in *radish*^1^ mutants and pharmacological rescue with methylphenidate demonstrate how the optomotor maze and tethered flight experiments can identify genetic and molecular determinants of attention span. Moreover, these assays can uncover the molecular basis of psychostimulant-mediated rescue in attention-like processes such as visual fixation, novelty response, and distractibility. However, with *radish*^1^ as the sole example of psychostimulant-mediated rescue of attention-like processes, these assays would benefit from validation using other known ADHD-linked mutants. The observation of similar deficits in ADHD-associated mutants, along with improvement following treatment with psychostimulants, would provide support for impaired responses in the optomotor maze and tethered flight experiments as endophenotypes for attention-like processes dysregulated in ADHD. Because ADHD is predominantly a polygenic disorder, with impairments occurring on a spectrum, it is unlikely that a single behavioral assay will be effective in studying every putative risk gene. The current models of ADHD-like behavior in flies address two broad features of ADHD: dysregulation of attention-like processes and dysregulation of arousal. Complementary use of the DAM system, optomotor maze, and flight loop experiments may be useful in unraveling how specific behaviors are improved with psychostimulant treatment, supporting the identification of genes and pathways that determine treatment efficacy.

Rare genetic variants associated with ADHD such as *LPHN3* and *NF1* have a large effect size, meaning a large portion of observed phenotypic variance can be attributed to these individual genes. In contrast, common genetic variants associated with ADHD have a smaller effect size, meaning their individual contribution to observed phenotypes is less severe. Rare disease variants, because of their increased severity, can be helpful in elucidating molecular mechanisms that can help understand the deficits observed in mild cases disease. However, identifying the common variants that contribute to mild-cases of diseases has been more difficult. Because these common variants have a small effect size, it is difficult to perform studies with enough subjects to reach the statistical power necessary to identify common risk genes. The first successful identification of common variant risk was performed in a 2019 GWAS that used data from more than 50,000 subjects to identify of 12 ADHD risk loci [136]. In contrast to human studies, *Drosophila* offer a model where additional genetic tools can circumvent the limitations associated with underpowered research. A recent study combined transcriptome and behavioral analysis across hundreds of methylphenidate exposed flies, to behavioral analysis of 172 lines from the DGRP to identify genetic variants that contribute methylphenidate response [137]. The study assigned 650,766 segregating single nucleotide polymorphisms (SNP) to 7472 gene networks and used an integrative genomic prediction approach to predict SNPs associated with differentially expressed transcripts. Of the 87 networks, including 1727 genes predicted to impact methylphenidate response, 14 of the top candidate genes were validated with RNAi using a ubiquitous GAL4 driver, and 10 of those genes altered response to methylphenidate. Twenty percent of the networks predicted to contain methylphenidate response genes are involved in histone modifying processes, and 4 of the 10 genes that were validated with RNAi are involved in histone modification [137], a process that is also relevant to human psychostimulant addiction [138]. This transcriptomic study demonstrates how *Drosophila* is a model that enables identification of novel candidate genes that mediate response to treatment with psychostimulant drugs, information that might also help elucidate differences in genetic etiology that contribute to treatment resistance in humans.

### 3.2. Autism Spectrum Disorder (ASD)

Most cases of ASD are polygenic and 80% of cases involve multiple genes, making it difficult to unravel how each gene contributes disease-related impairments [139]. Additionally, many patients with ASD also have comorbid ADHD, with estimates from clinical samples between ranging between 37–85% [140]. Flies are a useful model for studying the impact of individual genes associated with ASD [139]. The impact of several ASD-linked mutations on DAT function, reverse transport of dopamine, and sensitivity to amphetamine psychostimulants have been characterized in *Drosophila* [107,108,109].

Hamilton and colleagues used *Drosophila* to functionally characterize an ASD-associated de novo mutation in the human dopamine transporter (hDAT) at residue 356 (hDAT T356M). [107] Cell culture experiments showed hDAT-T356M caused constitutive reverse transport of dopamine and reduced amphetamine-induced reverse transport of dopamine. To identify how these changes impact behavior and response to psychostimulants, a transgene carrying the human ASD-associate DAT mutant was introduced into a *Drosophila DAT* knockout background (*DAT^fmn^*). hDAT-T356M expressing flies displayed increased baseline locomotor activity compared to control flies expressing hDAT. Additionally, while amphetamine increases locomotor activity in hDAT expressing flies, *Drosophila* expressing the ASD-associated mutant do not display an amphetamine-induced increase in locomotion [107].

A similar behavioral phenotype was observed in flies during an experiment involving the first characterization of an ASD-associated in-frame deletion in the human dopamine transporter (hDAT), which eliminates asparagine residue 336 (hDAT-ΔN336) [108]. Cell culture experiments performed to molecularly characterize hDAT-ΔN336 showed that the ASD-associated mutation stabilizes the transporter in a half-open and inward-facing conformation that disrupts DA uptake, but not reverse transport. To study the effects of this mutation in vivo, the ASD-associated *DAT* transgene was expressed in *DAT* knockout flies (*DAT^fmn^*), and compared to hDAT expressing controls. Flies expressing hDAT-ΔN336 displayed hyperactivity and increased grooming behavior compared to hDAT expressing control flies [108]. Flies expressing the mutant transporter also exhibited impaired amphetamine-induced reverse dopamine transport [108].

Another ASD-associated *DAT* mutant that impacts psychostimulant response contains an arginine to tryptophan substitution at N-terminal position 51 (hDAT-R/W), a residue important for interaction with the membrane protein Syntaxin-1A (STX1A) [109]. Murine models showed that the interaction of the DAT with STX1A support amphetamine-induced reverse transport of dopamine, and that STX1A overexpression increased reverse transport of dopamine [141]. Experiments in cell culture revealed that the ASD-associated hDAT-R/W did not affect DA uptake, but reduced amphetamine-induced reverse transport of DA [109]. *DAT^fmn^* flies expressing the ASD-associated variant were used to characterize how this mutation impacts behavior. No difference in basal locomotion was observed between hDAT-R/W expressing flies and hDAT expressing control flies. However, hDAT-R/W expressing flies exhibited a significant reduction in amphetamine-induced locomotion compared to hDAT expressing control flies [109].

These experiments demonstrate how flies be used to study the behavioral impact of mutations, linking the behaviors in question to molecular mechanisms characterized in cell culture. These experiments cannot be performed in humans, and can help characterize how specific mechanisms contribute to the pathogenesis of complex polygenic disorders such as ASD [107]. While these experiments analyzed psychostimulant response in the context of locomotion, flies have potential for modeling more complex behavioral features of ASD, which is known to impact social behavior. *Drosophila* display broad repertoire of social behaviors that range from population scale changes in group dynamics [142] to pairwise interactions involved in displays of aggression [143] and courtship [144]. Quantification of group density [145] and analysis of courtship behavior [89] are both established methods that have been used to study social behavior in *Drosophila,* and both respond to changes in dopamine. As social deficits are common in fly models of ASD, *Drosophila* could provide an effective model to study whether psychostimulant drugs can rescue social responses. These experiments could help identify specific genes and molecules required for psychostimulant-mediated improvements in social behavior.

## 4. Studying Psychostimulant Abuse with *Drosophila*

Addiction is an etiologically complex polygenic disorder that develops in an experience-dependent manner. In humans, features of addiction include increases in drug preference and consumption. Individual differences in sensitivity to the behavioral activating effects of psychostimulant drugs, the development of sensitization, initial preference, and patterns of drug consumption are all important factors predictive of future psychostimulant response and propensity to develop substance use disorder. Studies of psychostimulant-induced behavior in mammals show that simple locomotor responses can be predictive of more complex addiction-related behaviors such as the acquisition of self-administration. In rats, for example, sensitivity to the locomotor activating effects of cocaine predict the development of cocaine-conditioned place preference [146]. Here, we discuss the genes implicated in psychostimulant sensitivity, sensitization, and preference in *Drosophila*, and highlight their relevance in modeling psychostimulant addiction.

### 4.1. Using Drosophila to Study the Mechanism of Action of Psychostimulant Drugs

While the intoxicating effects of commonly abused psychostimulants vary in intensity and duration, they all act to increase dopaminergic signaling [41]. Dopamine is involved in many behaviors in *Drosophila*, including locomotion, attention-like processes, memory, and reward [147,148,149,150,151]. As dopamine is central to the mechanism of action for psychostimulant drugs, the genes involved in dopamine synthesis, neurotransmission, and dopamine receptor coupled signaling pathways directly impact psychostimulant response.

Tyrosine hydroxylase is the rate limiting enzyme involved in the conversion of L-tyrosine to dopamine [152]. RNAi-mediated knockdown of tyrosine hydroxylase in dopaminergic neurons of *Drosophila* larvae ablates locomotor response to amphetamine [106]. Tyrosine hydroxylase influences coloration of the flies’ cuticle, and loss of function mutants were named *pale* because of their discoloration [153]. *pale* mutants have reduced brain dopamine, are hypoactive [154,155], and do not exhibit a reduction in sleep after treatment with amphetamines [111]. Reducing dopamine also impacts response to cocaine, and flies fed 3-iodotyrosine (3IY), a competitive inhibitor of tyrosine hydroxylase show a reduction in cocaine-induced locomotion [58].

*dDAT* codes for the *Drosophila* plasma membrane dopamine transporter (DAT) that functions to remove extracellular dopamine from the synaptic cleft [156]. DAT structure, function, and interaction with psychostimulants are highly conserved in *Drosophila* and humans [38,40]. In *Drosophila,* the activating effects of psychostimulants on arousal are dopamine transporter dependent. Both amphetamine and methylphenidate increase locomotion in *Drosophila* larvae, a response that is blunted in *DAT^fmn^* flies or flies expressing *dDAT* RNAi in dopaminergic neurons. Expression of the human dopamine transporter (hDAT) in *DAT^fmn^* mutants rescues the hyperlocomotive response to both amphetamine and methylphenidate [106], demonstrating that a conserved DAT-dependent mechanism facilitates the locomotor-activating effects of psychostimulant drugs. Consistent with the data showing that response to psychostimulant drugs is contingent on the presence or absence of functional DAT, sensitivity or resistance to psychostimulants drugs is also dependent on DAT cell surface expression [157]. *Drosophila Ric*, encodes a Ras-related small GTPase involved in dopamine transporter trafficking [158]. *Rit2*, the mammalian orthologue of *Ric*, has been shown it impact cocaine sensitivity in mice [159]. In *Drosophila*, dopaminergic expression of a constitutively active Ric-GTPase mutant (*RicQ117L*) increases cell surface expression of DAT and enhances sensitivity to the locomotor activating effects of amphetamine [157].

While the activating effects of cocaine, methylphenidate, and amphetamine-like psychostimulants are all DAT-dependent, they do not increase dopaminergic signaling through the same mechanism of action (Figure 1). Cocaine as well as methylphenidate act as competitive inhibitors of the dopamine transporter [160] that increase dopaminergic signaling by binding *dDAT* and reducing uptake of synaptic dopamine [161], while amphetamine-like psychostimulant bound to the dopamine transporter activate reverse transport of dopamine [43,162,163]. In vitro studies first showed the reverse transport of dopamine stimulated by amphetamine-like psychostimulants was regulated by Calcium/calmodulin-dependent protein kinase II (CaMKII) mediated phosphorylation of the DAT the N-terminal domain [152,153] and localization of the DAT to specific plasma membrane microdomains by the membrane lipid raft protein flotillin-1 [164]. The first in vivo experiment to show that flotillin-1 is required in dopamine neurons for amphetamine-induced locomotion was performed in *Drosophila* [106]. In contrast to the increased locomotion observed in wild-type flies, *Flotillin 1* mutant (*Flo1^02554^*) larvae or larvae expressing *Flo1* RNAi in dopamine neurons did not display an increase in locomotive speed after administration of amphetamine [106]. Expressing human *FLOT1* in dopaminergic neurons of *Flo1^02554^* mutant larvae rescued the hyperlocomotive response to amphetamines [106]. One explanation for the requirement of *Flo1* in amphetamine-induced reverse transport might be the role of lipid rafts in acting as a scaffold for other signaling proteins such as CamKII [165,166]. In vitro experiments showed that amphetamine-induced reverse transport depends on CamKII-mediated phosphorylation of DAT N-terminal serine residues (2, 4, 7, 12 and 13), a modification associated with the inward-facing transporter conformation that facilitates reverse transport [162]. This dependency was confirmed in vivo using *Drosophila DAT^fmn^* mutants, where dopaminergic expression of an *hDAT* construct with serine to alanine substitutions that prevent phosphorylation ablates amphetamine-induced hyperlocomotion [106]. The hyperlocomotive response to amphetamines is rescued by dopaminergic expression of wild-type *hDAT*, as well as a mutant *hDAT* where N-terminal serine residues are replaced with phospho-mimetic aspartate residues. Flies were also used to demonstrate DAT phosphorylation requires CAMKII in dopamine neurons, and that expressing CaMKII inhibitory peptide in the dopamine neurons of larvae ablates amphetamine-induced hyperlocomotion [112]. Dopaminergic expression of the N-terminal domain phosphomimic *hDAT* mutant rescues the locomotor response to amphetamine in flies expressing CaMKII inhibitory peptide [112]. *Drosophila* were also the first model organism to show that the membrane phospholipid Phosphatidylinositol (4,5)-bisphosphate (PIP2) interaction with the human dopamine transporter is important for amphetamine-induced dopamine efflux [110]. In silico experiments predicted that DAT N-terminal lysine residues 3 and 5 were involved in interaction with PIP2. In vitro experiments demonstrated that neutralizing alanine substitutions lysine residues 3 and 5 (*hDAT-K/A*) do not alter DAT cell surface expression, but to reduced amphetamine-induced dopamine efflux. To test the behavioral impact of the hDAT-K/A mutation, *DAT^fmn^* mutant flies expressing hDAT or hDAT-K/A in dopaminergic neurons were fed amphetamine and monitored for changes in locomotor behavior. While hDAT expressing flies fed amphetamine showed an significant increase in locomotion, this effect was abolished in hDAT-K/A expressing flies [110]. The DAT lysine at residue 337 and arginine 443 in intracellular loop 3 and 4 were also predicted to interact with PIP2 based on computational modeling experiments [110]. In vitro experiments showed that neutralizing alanine substitutions at *K337* and *R443* lead a reduction in DAT cell surface expression of more than 80% [110]. To test the behavioral impact of the arginine to alanine substitution at residue 443 (*hDAT-R443A*), which did not affect surface localization, *DAT^fmn^* mutant flies expressing hDAT or hDAT-R443A in dopaminergic neurons were observed in the DAM system. No difference was observed when comparing baseline locomotion of hDAT and hDAT-R443A expressing flies. However, a difference in locomotion was observed after exposure to 1 mM amphetamine, and *DAT^fmn^* flies expressing *hDAT* displayed an increase in cumulative locomotion while no significant change was observed in *DAT^fmn^* flies expressing hDAT-R443A [91]. In addition to impacting locomotion, the interaction of PIP2 with DAT residue 443 is required for amphetamine preference in a CAFE with the choice between sucrose and sucrose supplemented with amphetamine. Preventing the interaction of PIP2/DAT in *DAT^fmn^* flies expressing hDAT-R443A abolishes preference of 1 mM amphetamine that is observed in *DAT^fmn^* flies expressing wild-type hDAT. In contrast to the preference observed at low doses of amphetamine (1 mM), *DAT^fmn^* flies expressing hDAT avoid amphetamine at higher doses (10 mM amphetamine). *DAT^fmn^* flies expressing hDAT-R337A also display avoidance for 10 mM amphetamine in the CAFE, suggesting the interaction of PIP2 with DAT is required for amphetamine preference, but not amphetamine avoidance [91].

The vesicular monoamine transporter 2 (*VMAT2*) regulates the storage and release of dopamine, and is a key mediator of behavioral responses to psychostimulants of abuse [167]. The *Drosophila* homologue of *VMAT2* (*dVMAT*) has two splice variants, with dVMAT-A identified to play a role in dopamine transport similar to VMAT2 [168]. In flies, overexpression of dVMAT-A in *Drosophila* dopamine neurons leads to increased release of DA, along with an increase in grooming behavior, an increase in locomotion, and a decrease in negative geotaxis [71]. Similar increases in grooming behavior and locomotion are observed in wild-type flies after 5–7 days of cocaine administration, as well as control flies that do not overexpress the *dVMAT-A* transgene. Interestingly, flies that overexpress the *dVMAT-A* transgene in dopaminergic neurons display a decrease in behavioral sensitivity to cocaine in assays of locomotion, grooming, and negative geotaxis. One explanation for this blunted response to cocaine might be a ceiling effects related to the overexpression of dVMAT-A, suggesting that cocaine mediated increases in synaptic dopamine are behaviorally inert in a system that is already saturated with dopamine. This explanation does not, however, justify the response to acute cocaine exposure in flies overexpressing dVMAT-A, where increasing dVMAT-A expression actually reduced the sensitivity to the impairing effects of cocaine on negative geotaxis [71]. This effect was dose-dependent: flies with more copies of the *dVMAT-A* transgene displayed the greatest reduction in cocaine induced impairment [71]. These experiments highlight the utility of *Drosophila* in uncovering molecular interactions that cannot be determined in humans. Such experiments might be especially useful for identifying the behavioral effects VMAT2 polymorphisms associated with an increased risk of addiction [169,170].

In contrast to flies overexpressing dVMAT-A, mutant flies with a null mutation of *dVMAT* show a decrease in dopamine levels compared to wild-type flies, with measurement of dopamine in whole heads from flies heterozygous and homozygous for null mutation containing 35% and 75% of normal dopamine levels, respectively [72]. Surprisingly, opposite responses were observed in flies heterozygous and homozygous for the null mutation in the context of larval locomotion, negative geotaxis, and dark reactivity, with heterozygotes displaying an increase larval locomotion, and adult climbing behaviors and homozygotes displaying a decrease in the same behaviors [72]. In adult *Drosophila*, both heterozygotes and homozygotes displayed an increase in baseline locomotor activity compared to wild-type flies. Additionally, while cocaine produced a large increase in locomotor activity in wild-type flies, only a small increase in locomotion was observed in heterozygous flies, and no significant change was observed in flies homozygous for the null mutation [72]. VMAT is also important in the context of behavioral response to amphetamine. Wild-type as well as *dVMAT* null *Drosophila* larvae display an increase in locomotion in response to amphetamine, however, that response is diminished by 5-fold in *dVMAT* null mutants [59]. VMAT is an established therapeutic target and changes in VMAT function are known to influence responses to drugs of abuse [171], making it a relevant molecule for studying response to psychostimulant drugs in *Drosophila.*

All psychostimulants of abuse act to increase synaptic dopamine, and their locomotor activating as well as rewarding effects similarly depend on the activation of dopamine receptors along with their downstream signaling pathways. In *Drosophila*, there are two different D1-like dopamine receptors (Dop1R1 and Dop1R2), one D2-like dopamine receptor (DD2R), and one dopamine and ecdysone hormone receptor (DopEcR) [172]. *Drosphila* dopamine receptors are integral to the effects of psychostimulants on arousal [173], locomotion [76], and reward [60]. In wild-type flies, methamphetamine [173] and cocaine [76] increase arousal and reduces sleep. The sleep reducing effects of methamphetamine are lost in *dumb*^1^ mutant flies, where Dop1R1 expression is significantly reduced. Expressing Dop1R1 in the mushroom body of *dumb*^1^ mutant flies restores the methamphetamine-induced reduction in sleep [173]. Dop1R1 expression is also significantly reduced *dumb*^2^ mutant flies, who show no reduction in sleep after cocaine exposure [76]. *dumb*^2^ mutant flies also display an increase in repetitive startle induced hyperactivity (ReSH), where active flies subjected to repeated bouts of mechanical stimulation display an increase in locomotion and a protracted period of hyperactivity greater than that observed in wild-type flies [76]. Interestingly, while cocaine increases arousal of wild-type flies in the context of sleep, cocaine decreases the arousal associated with ReSH. This effect is not observed in *dumb*^2^ mutant flies, where ReSH is the same in the presence and absence of cocaine [76].

In addition to regulating psychostimulant-induced arousal, *Drosophila* dopamine receptors regulate psychostimulant preference, and mushroom body knockdown of Dop1R1 or Dop1R2 both suppress acute methamphetamine preference in the CAFE [60]. In contrast, DopEcR mutants display an increase in acute preference for methamphetamine [60]. These results highlight the dynamic nature of dopaminergic signaling in driving appetitive as well as aversive responses, and demonstrate how dopamine receptor subtypes modulate psychostimulant preference. Dopamine receptors play a similar role in the modulation of preference in humans, where changes in receptor expression are associated with drug craving and drug seeking behavior [174]. Identifying the specific dopaminergic circuits that mediate psychostimulant preference or avoidance in *Drosophila* will aid in discovery of the genes that modify each pathway, and bias behavior for or against the development of addiction.

### 4.2. Using Drosophila to Identify Novel Genes Involved in Response to Psychostimulant Drugs

While *Drosophila* have been useful in molecular characterization of genes known to impact the psychostimulant response in mammals, they have also been instrumental in identifying novel genes that regulate response to psychostimulant drugs. Research in flies was the first to show that mutation of the circadian gene *period* increased initial sensitivity to the motor activating effects of cocaine, but disrupted the development of sensitization [63]. The role of circadian genes *clock*, *cycle*, and *doubletime* were also identified in mutant flies that failed to develop sensitization to the motor activating effects of cocaine [63]. Subsequent studies confirmed the role of *Period* [175] and *Clock* [176] in response to cocaine in mammals. Additional experiments in *Drosophila* also demonstrated that *period* is involved in the regulation of dopamine receptor responsiveness [177]. Additionally, while wild-type flies develop a preference for methamphetamine in the CAFE, *period* null mutants do not develop preference and do not self-administer methamphetamine [69].

*Drosophila* were also used to identify a novel role of *Lim-only (dLmo)* gene in modulating response to cocaine [86]. The expression of *dLmo* regulates the sensitivity of primary pacemaker neurons to cocaine-induced increases in synaptic dopamine, with loss of function mutants exhibiting an increase in sensitivity to the motor-activating effects of cocaine on locomotion and the motor-impairing effects of cocaine on negative geotaxis [86]. In contrast, *dLmo* gain of function mutants are resistant to the effects of cocaine, and display a reduction in cocaine-induced locomotion and a decrease in impairment of negative geotaxis [86]. While the relationship between dysregulation of circadian rhythms and substance abuse is now well established in mammals [178], the initial experiments demonstrating of role of circadian genes in modulating response to drugs of abuse were performed in flies. These experiments highlight the value of *Drosophila* as a translational genetic model for identifying novel regulators of drug response that can inform our understanding of human disease.

Protein kinase (PKA) is involved in signaling molecular targets downstream of the *Drosophila* circadian pacemaker cells [179]. The *Drosophila* type II cAMP-dependent protein kinase (PKA-RII) is also involved in circadian regulation, and acts downstream of circadian pacemaker cells. PKA mutants display reduced sensitivity to the motor activating effects of cocaine in the behavioral scoring assay, require more than double the amount required to generate a response in control flies, and do not develop sensitization to repeat exposures [116]. D1 receptor-mediated PKA signaling is also involved in the expression of behavioral sensitization to cocaine in rats [180], and chronic cocaine administration in mammals is associated with dopamine receptor-mediated activation cAMP and PKA signaling cascades involved in the development of addiction [174]. Several components of the cAMP and PKA signaling pathway have demonstrated success as drug target, and *Drosophila* are a promising model organism to help identify potential druggable targets that might be useful in the development of therapeutics for the treatment of addiction [181]. 

In addition to PKA, several other molecules associated with regulating synaptic plasticity have also been shown to impact psychostimulant response in flies. The Rho family GTPase activating protein RhoGAP18B regulates actin dynamics and response to alcohol in *Drosophila*, and several *whir* mutants have reduced sensitivity to the motor impairing effects of cocaine on negative geotaxis behavior [77]. The Ste20 family kinase Tao is also a regulator of cocaine response in flies, and *tao* mutants are resistant to the motor-impairing effects of cocaine on negative geotaxis [182]. The *Drosophila* GSK-3β homologue *shaggy* is another molecule implicated in the regulation of synaptic plasticity that is immediately downstream of PKA, and regulates the activity of multiple proteins including cAMP-response element binding protein (CREB), timeless, and the microtubule-binding protein tau [183]. The *shaggy^EP1379^* mutant, missing the serine 9 site regulated by PKA, produces flies sensitive to cocaine-induced effects on negative geotaxis [184]. While the mechanisms of these genes in regulating psychostimulant response remain to be determined, there is evidence of psychostimulant regulation by analogous genes in mammals. Rho family GTPases are G-proteins that regulate actin dynamics [185], and are involved in the locomotor response to amphetamines [186] and the development of cocaine preference [187] in mice. *TAOK1*, the mammalian orthologue of *tao*, is transcriptionally regulated in mammalian models of cocaine and methamphetamine addiction [188,189]. GSK-3β is involved in the development of behavioral sensitization to the locomotor activating effects of cocaine in rats [190], and cocaine withdrawal disrupts the circadian regulation of GSK-3β [191]. Knockout of *Tau* enhances cocaine conditioned place preference in mice while Tau overexpression in the hippocampus suppresses cocaine conditioned place preference [192]. The identification of parallel pathways for the regulation of psychostimulant response in flies and mammals has implications for the identification of novel regulators of psychostimulant response. Unbiased screens identifying genes that modify alcohol consumption in flies have been successfully translated to identify the genes that regulate alcohol consumption in humans [193,194]. Additionally, *Drosophila* provide the ability to test hypotheses related to the development of targeted therapies for addiction, which is specifically relevant for Rho family GTPase and GSK-3β signaling pathways which are promising drug targets for the treatment of neuropsychiatric disorders [195,196].

While the assays used to measure psychostimulant response in *Drosophila* do not completely recapitulate the features of psychostimulant abuse in humans, they effectively capture features of drug response that can be used to identify novel genes that predict the risk to develop addiction. While there is currently no *Drosophila* equivalent of the operant-controlled psychostimulant self-administration paradigms commonly used in rodent models of addiction, such as the lever press, analysis of simple behaviors such as voluntary consumption are an equally effective measure of hedonic value [197]. The decrease in complexity of behavioral assays in flies is directly related to the increase in speed, simplicity, and throughput of functional genetic experiments that make *Drosophila* such a powerful model organism. The benefits of this model are especially relevant to the study of complex polygenic disorders such as addiction, which are difficult to study in human populations. While the heritability of risk genes for the development of addiction has been established with family studies and genome-wide association studies (GWAS), it is difficult to collect enough human data to identify all of the genes involved in psychostimulant abuse. In addition solving the problem of sample size, tools such as the *Drosophila melanogaster* Genetic Reference Panel (DGRP) enable characterization of the relationship between genetic variation and quantitative traits [198]. The DGRP is used for mapping quantitative trait loci (QTLs) associated with various psychostimulant phenotypes among a population of 192 inbred strains that provide a representative pool of naturally occurring genes [198].

The DGRP has been used to study the genetic basis of variation in cocaine and methamphetamine consumption in a 2-choice CAFE [93]. Voluntary consumption, preference, and experience-dependent changes in voluntary consumption or preference were found to be significantly influenced by large polygenic gene networks both distinct and shared between cocaine and methamphetamine. Specific groups of genes were identified in relation to consumption, preference, and the effect of exposure on consumption and preference. Genes underpinning variation in responses related to sexual dimorphism were also identified. Additionally, analyses of consumption behavior for each drug were performed to identify interactions between exposure, sex, and solution preference. A total of 1358 candidate genes were identified across all networks, with a significant network of 81 shared candidate genes for all traits combined [93]. The network of candidate was validated with a series of RNAi experiments. First, in 34 selected candidate genes with a weak, ubiquitous GAL4 driver, which produced an effect on at least one measure of consumption for each gene. Then again, using neuron and glial specific drivers, to knockdown the 10 genes that displayed the strongest phenotype among the 34 analyzed in the first round of RNAi. All of the tested genes had a significant effect on at least on measure of consumption, highlighting the capability of *Drosophila* in effectively identifying genes the determine consumption and preference for psychostimulant drugs [93].

A similar experiment was performed to identify the genetic basis of variation in cocaine and methamphetamine consumption in single-fly CAFE assays with 18,000 individuals flies generated from an outbred advanced intercross population (AIP) derived from 37 highly diverse, fully sequenced inbred lines from the DGRP [92]. This design maximizes genetic variability to provide more precise estimates of QTL. In contrast to the previous screen, flies in this assay were only offered one feeding solution, restricting analysis to voluntary consumption, and the change in voluntary consumption over time. Similar analyses were performed to identify gene networks associated with phenotypic difference in consumption, relative change in consumption over time, and variation in responses related to sexual dimorphism. A total of 1962 candidate genes were identified, and 22 were validated using RNAi, 17 of which influenced consumption of methamphetamine or cocaine. In addition to plausible technical issues such as functional redundancy or weakness of the driver for RNAi, one explanation for the lack of effect in all 22 genes could be the inability of candidate-based RNAi validation to recapitulate variability that might arise from intergenic regions of DNA. Therefore, a single nucleotide polymorphism-based validation was used to isolate the two alleles from the AIP that caused high and low consumption, respectively, in a shared genetic background to test the effect of intragenic and intergenic, naturally occurring SNPs on consumption. Using this method, an effect was observed for all but one candidate SNP. This not only highlights the power of *Drosophila* in performing unbiased screens genes that regulate psychostimulant consumption, but also demonstrates the ability to identify the contribution of common intergenic SNPs—analogous to many human SNPs associated in various GWAS analyses—to phenotypic variation.

One question that is not possible to investigate in human studies is what genes are transcriptionally regulated in response to acute psychostimulant drug exposure. Analysis of single-cell transcriptional responses in psychostimulant-exposed flies enables investigation of this question at scale and resolution that is not feasible in other models organisms. To characterize the impact of cocaine on transcriptional response, 114 female along with 128 male flies were selected for analysis after ingesting equal amounts of cocaine solution, and similar number of control flies were selected after ingesting an equivalent volume of sucrose [199]. The acute behavioral effects of cocaine were analyzed by measuring effects on locomotion, impairment of negative geotaxis, startle response, and seizure activity. An analysis of single-cell transcriptional responses revealed 691 and 322 differentially expressed genes in males and females, respectively. These genes were segregated into 36 distinct clusters based on expression profiles. The results indicate the single-cell transcriptional response to cocaine is sexually dimorphic, influences a large range of cellular processes, induces significant responses in neurons of the mushroom body to include transcription of signaling genes as well as regulators of dopaminergic neurotransmission, and impacts transcription of several genes in glia including those involved in blood–brain barrier regulation. Importantly, different clusters of brain cells reveal distinct suites of cocaine-regulated genes, indicating cell-type specific consequences of acute drug exposure. Sixty-nine percent of the genes involved in the transcriptional response to cocaine in *Drosophila* have human orthologues, and several of the identified genes are known to play a role in cocaine response in humans [199]. The identification of genes with an established role in reward, addiction, and cocaine response in humans highlights the potential for identifying novel genes that are transcriptionally regulated by psychostimulants of abuse using *Drosophila*.

With the increasing accessibility of high-throughput sequencing techniques, multiple methods of genetic analysis might be combined to identify novel genes involved in response to psychostimulant. The recent experiments using AIPs generated from inbred lines of the DGRP exemplify the capability of *Drosophila* in unveiling novel regulators psychostimulant response, demonstrating how flies can be used to identify the genes that regulate psychostimulant response in complex polygenic disorders such as addiction and ADHD [90,91]. These screens are leading examples of the speed and throughput *Drosophila* offers, enabling analyses that would be prohibitive in a mammalian model organism. Similar utility is showcased in analysis of single-cell transcriptional response to cocaine [199]. While these studies are leading the field in characterizing the genetic basis of psychostimulant response, they are the only examples of high-throughput screens for novel genes, and there is significant opportunity for future replication and mechanistic investigation.

## 5. Future Directions

*Drosophila* is in the evolving stages as a model for studying the biological basis of response to psychostimulant drugs of abuse, but has unparalleled potential for identifying novel genes and molecules with an efficiency that could not be achieved in other models. Genetic and behavioral accessibility of *Drosophila* contribute to flexible manipulation of genes in high-throughput experiments that recapitulate many of the complex behavioral responses observed in humans. There are over 200 human disease models in *Drosophila,* including diseases that respond to psychostimulant treatment such as ADHD and ASD. Additionally, *Drosophila* is a powerful translational model for studying addiction, and have proven successful in identifying genes that regulate alcohol consumption in humans. In contrast to the substantial amount of research on AUD in flies, there are still relatively few studies on psychostimulant response. The majority of psychostimulant experiments in *Drosophila* focus on sensitivity to motor-activating or motor-inhibiting drug effects, and the development of behavioral sensitization. While these assays of sensitivity and sensitization have been used for more than 20 years [62,63], studies involving self-administration are relatively new. Regarding studying of the effects of psychostimulants on attention-like processes, the optomotor maze along with the flightless arena are significantly involved and low throughput. There is, therefore, an opportunity window to optimize the current assays to improve the study of psychostimulant responses in *Drosophila*.

In the context of addiction, several newer assays of consumption and preference such as the Fly Liquid-Food Interaction Counter [200] and Fly Liquid-Food Electroshock Assay (FLEA) [201] provide improvements in automation for acquisition as well as analysis of behavior, enabling comprehensive tracking of experience dependent changes in response. Both the FLIC and the FLEA support high-throughput continuous monitoring of behaviors indicative of consumption and preference. Additionally, the FLEA supports the pairing of a punishing electric shock with a food source, which allows analysis of persistence of voluntary consumption or preference in the presence of aversive stimuli. This experimental design provides an opportunity to model important features of human addiction such as continued drug use in spite of negative consequences. The punishing shock could also be used to examine drug-induced changes in delay-discounting, another endophenotype important for understanding substance abuse.

These assays can also be used to investigate the mechanisms of psychostimulant response in ADHD models. While psychostimulant medication is effective in ameliorating the cognitive and behavioral deficits associated with ADHD, the mechanism of this behavioral response is poorly understood. The unbiased generation of candidate genes that impact psychostimulant response from screens using AIPs of the DGRP [92,93] provide a pool of putative genes that might also contribute the pathogenesis of ADHD. Similarly, an analysis of single-cell transcriptional responses to both cocaine [199] and methylphenidate [17] enables clustering of transcriptional profiles that could provide information on the circuits and pathways are activated by psychostimulant drugs in wild-type flies. Replicating the same experiment with mutant *Drosophila* that display behavioral features of ADHD [101,105,121] would allow comparison of transcriptional responses and the clustering of transcriptional profiles comparing ADHD-related and control flies. Analysis of changes in transcriptional response between ADHD-phenotypes might provide correlative data associating phenotype severity with clustering of transcriptional profiles. This representation could provide an innovative way to identify disease related patterns of transcriptional regulation. This data might be especially relevant for disorders such as ADHD and ASD, where deficits occur on a spectrum, with some impairments resulting from cumulative deficits between and within neurological circuits.

In examining the use of *Drosophila* to study behavioral responses to psychostimulant drugs we have shown several ways that flies can provide new insights into the biological basis of psychostimulant addiction. Additionally, we have reviewed how *Drosophila* is an effective model organism for identifying genes that impact the therapeutic efficacy of psychostimulant drugs. Together, the implementation of improved behavioral assays combined with high-throughput next generation analysis methods will help identify the distinct gene networks and mechanisms that mediate psychostimulant responses in *Drosophila* models of ADHD, ASD, and substance abuse. Due to the translatability of *Drosophila* research, these experiments have the potential to uncover novel druggable targets relevant for human therapeutics.

## Figures and Tables

**Table 1:** Genes involved in *Drosophila* psychostimulant response.

Gene	Homologue ^1^	Gene Function ^2^	Mutant ^3^	SUD Related Behavior	Psychostimulant Response ^4^	Disease Model
*iav*	*TRPV6*	ion channel	LoF	sensitization	mutants do not sensitize to COC [62]	
*Dop1R1*	*DRD1, DRD5*	DA signaling	KD	consumption, preference	MB KD alters experience dependent change in consumption of COC and MA [93]	
			LoF, KD	consumption, preference	mutation or MB KD disrupts acute and experience dependent MA preference [60]	
*Dop1R2*	*ADRB1*	DA signaling	LoF, KD	consumption, preference	reduced preference for MA [60]	
*Dop2R*	*DRD2*	DA signaling	null	consumption, preference	reduced preference for MA [60]	
*DopEcR*	*GPR21*	DA signaling	null	consumption, preference	increased preference for MA [60]	
*DAT*	*DAT1*	DA reuptake	null	locomotion	*dDAT^fmn^* flies do not exhibit hyperlocomotive response to AMPH [106]	
			partial LoF	locomotion	*DAT^fmn^* flies expressing *hDAT-T356M* have blunted locomotor response to AMPH [107]	ASD
			partial LoF	locomotion	*DAT^fmn^* flies expressing *hDAT-ΔN336* are hyperactive and have impaired AMPH -induced reverse DA transport [108]	ASD
			partial LoF	locomotion	*DAT^fmn^* flies expressing the ASD-associated variant *hDAT-R/W* display a decrease in AMPH-induced locomotion [109]	ASD
			partial LoF	locomotion	*DAT^fmn^* flies expressing *hDATK/A* have blunted locomotor response to AMPH [110]	
			partial LoF	locomotion	*hDAT-R443A* mutants have a blunted locomotor response to AMPH [91]	
			partial LoF	consumption, preference	*hDAT-R443A* mutants do not develop preference in the CAFE [91]	
			KD	sleep, arousal	MPH rescues sleep deficit in DAT pan-neuronal KD [105]	ADHD
			null	sleep, arousal	AMPH decreases hyperactivity and induces sleep in *DAT^fmn^* flies [111]	ADHD
*CaMKII*	*CAMK2D*	cell signaling	expression of inhibitor	locomotion	dopaminergic expression of CaMKII inhibitor abolishes AMPH-induced hyperlocomotion [112]	
*Flo1*	*FLOT1*	membrane protein	LoF	locomotion	*Flotillin 1* mutants *(Flo^e02554^*) have a blunted locomotor response to AMPH [106]	
*dVMAT*	*VMAT2*	MOA transport	OE	motor- impairment	OE decreases COC-induced impairment of negative geotaxis [71]	
			OE	locomotion	OE blunts COC-induced increases in locomotion [71]	
			null	locomotion	reduced locomotor response to COC [72]	
			null	locomotion	reduced locomotor response to AMPH [59]	
			pharmaco- logical inhibition	locomotion	VMAT2 inhibitor reduces COC-induced motor activation [58]	
*ple*	*TH*	DA biosynthesis	null	locomotion	*ple* flies do not exhibit AMPH-induced increases in locomotion [106]	
		DA biosynthesis	partial KO	locomotion	TH-deficient files have a blunted locomotor response to AMPH [111]	
		DA biosynthesis	targeted silencing, or activation	attention-like processes	acute MA exposure rescues optomotor response in flies expressing UAS-tnt or a truncated potassium channel (UAS-eag^Δ932^) in DA neurons [113]	
*LMO*	*LMO1*	circadian regulation	GoF	motor- impairment	mutants are resistant to COC-induced impairment of negative geotaxis [86]	
			null, partial LoF	motor- impairment	mutants have increased sensitivity to COC-induced impairment of negative geotaxis [86]	
*dbt*	*CSNK1D/E*	circadian regulation	hypmorph, hypemorph	motor- activation	mutants have reduced sensitivity to initial COC exposure, and do not sensitize to repeated exposures [63]	
*per*	*PER3*	circadian regulation	null,	motor-activation, motor- impairment	mutants are sensitive to initial COC exposure, but do not sensitize to repeated exposures at any dose [63,67,87]	
			hypmorph, hypemorph	motor-activation	short and long period mutants display increase in behavioral score for initial COC exposure, but display limited sensitization to repeated exposures [63]	
			null	sensitization	null mutants do not develop locomotor sensitization to vaporized MA [69]	
			null	consumption	mutants do not self-administer MA [69]	
*Pdf*	NA	circadian regulation	null	sensitization	mutants fail to develop sensitization to COC [68]	
*dClk*	*CLOCK*	circadian regulation	hypomorph	sensitization	mutants are less likely to develop sensitization to COC [68]	
*cyc*	*BMAL1*	circadian regulation	LoF	sensitization	mutants are less likely to develop sensitization to COC [68]	
*tim*	*TIM*	circadian regulation	LoF	locomotion	mutants have increased sensitivity to COC [68]	
*msi*	*MSI2, MSI1,*	development	targeted KD	consumption	MB KD increases COC preference [93]	
*Snoo*	*SKI; SKIL*	development	targeted KD	consumption, preference	MB KD increases initial COC preference in males and decreases initial MA preference in females [93]	
*ed*	*NPHS1*	development	targeted KD	consumption, preference	MB KD increases initial MA preference in males, and decreases experience dependent MA preference in males and females [93]	
*NA*	*APP* *; BACE1*	dysregulated in NDD	targeted expression	sleep, arousal	pan-neuronal expression of AβPP and hBACE1 produce ADHD-like phenotype rescued by MPH [114]	ADHD
*Cirl*	*LPHN1*	cell adhesion, signaling	KD	sleep, arousal	methylphenidate rescues ADHD-like behavior in pan-neuronal knockdown [105]	ADHD
*Nf1*	*NF1*	GTPase activation	KD	sleep, arousal	MPH rescues ADHD-like behavior in pan-neuronal knockdown [105]	ADHD
*moody*	*GPR84*	BBB permeability	partial LoF	motor- impairment	increased sensitivity to COC-induced impairment of negative geotaxis [115]	
*pika-RII*	*PRKAR2A*	cAMP signaling	severe LoF/null	motor-activation	reduced sensitivity to the motor-activating effects of COC; no sensitization to repeated exposure [116]	
*whir*	*ARHGAP9*	GTPase activation	LoF	motor- impairment	resistant to the motor-impairing effects of COC on righting behavior [77]	
*radish*	*GARNL3*	synaptic morphology, memory	LoF	attention-like processes	MPH rescues optomotor response, response to novel visual stimuli, and hyperactivity [101]	ADHD
*Rab10*	*RAB10*	GTPase	DN-Rab10	locomotion	pan-neuronal expression of DN-Rab10 reduces MA-induced locomotion and MA-induced mortality [117]	

^1^ Human orthologues of *Drosophila* genes involved in psychostimulant response. ^2^ Gene function abbreviations include dopamine (DA), monoamine (MOA), neurodegenerative disease (NDD), blood brain barrier (BBB), dominant negative (DN). ^3^ Mutant description abbreviations include gene knockout (KO), loss of function (LoF), gain of function (GoF), knockdown (KD), partial knockdown (pKD), overexpression (OE). ^4^ Abbreviations in psychostimulant response column include cocaine (COC), amphetamine (AMPH), methamphetamine (MA), and methylphenidate (MPH), tetanus-toxin light chain (UAS- tnt), mushroom body (MB), blood brain barrier (BBB), human platelet amyloid-β protein precursor (AβPP), and Beta-secretase 1 (hBACE1).

## Data Availability

Not applicable.
